# Editorial: Human-centred computer audition: sound, music, and healthcare

**DOI:** 10.3389/fdgth.2023.1340517

**Published:** 2023-12-12

**Authors:** Kun Qian, Gyorgy Fazekas, Shengchen Li, Zijin Li, Björn W. Schuller

**Affiliations:** ^1^ Key Laboratory of Brain Health Intelligent Evaluation and Intervention (Beijing Institute of Technology), Ministry of Education, Beijing, China; ^2^ School of Medical Technology, Beijing Institute of Technology, Beijing, China; ^3^ Centre for Digital Music (C4DM), School of Electronic Engineering and Computer Science, Queen Mary University of London, London, United Kingdom; ^4^ Department of Intelligent Science, School of Advanced Technology, Xi’an Jiaotong-Liverpool University, Suzhou, China; ^5^ Department of Music AI and Music Information Technology, Central Conservatory of Music, Beijing, China; ^6^ GLAM – the Group on Language, Audio, & Music, Imperial College London, London, United Kingdom; ^7^ Department of Computer Science, Embedded Intelligence for Health Care and Wellbeing, University of Augsburg, Augsburg, Germany

**Keywords:** computer audition, machine learning, deep learning, artificial intelligence, brain sciences

**Editorial on the Research Topic** Human-centred computer audition: sound, music, and healthcare

## 1. Introduction

At the time of writing this editorial, OpenAI has announced its newest model called chatGPT-4 Turbo.^1^ When dreaming for the blue print that we can better the life via this revolution of AI technologies by foundation models, it is a time for almost every person to think how to live with the powerful artificial intelligence (AI) models in the future.

A future that may also challenge our societies and current living in many ways (1) including or even particularly in healthcare (2). Thinking especially of audio, a similar rise of increasingly capable and powerful foundation models appears at highly accelerated pace and with increasingly emergent behaviour. One of the latest at the time of writing is Uniaudio—showing an overly impressive range of zero-shot abilities (3).

For a long time in the field of health, machines have been taught to “see” and/or to “read” rather than to “listen.” This is one of the reasons why more progress was achieved in the field of computer vision (CV) and natural language processing (NLP) rather than computer audition (CA) in this domain. Nevertheless, the promising contributions of audio cannot be ignored for its excellent performance in healthcare (4).

Motivated by the concept of human-centred AI (HAI), we organised the research topic on “Human-Centred Computer Audition: Sound, Music, and Healthcare,” which lasted from April 2021 to January 2023. Finally, 10 articles were accepted and published after a rigorous peer-review process. There are 57 authors involved in this research topic.

In the remainder of this editorial, we will briefly introduce the published research articles in this research topic collection. Then, insights and perspectives will be given towards the future work.

## 2. Contributions

The published contributions have covered the planned scope, e.g., computational analysis of sound scenes and events, digital music, computer audition for healthcare, computational paralinguistics, and explainable AI in computer audition. In the following, grouped by categories, we provide a brief description of the collected articles.

### 2.1 Fast screening of COVID-19

Whether audio could serve as a novel digital phenotype for detection of COVID-19 has been increasingly studied in the past three years (5, 6). Coppock et al. summarise the contributions in the organised INTERSPEECH 2021 Computational Paralinguistics Challenges: COVID-19 Cough, (CCS) and COVID-19 Speech, (CSS) (7). They indicated that, a classifier trained by the infected individuals’ respiratory sounds can achieve moderate detection rates of COVID-19. However, whether the audio biomarkers in respiratory sounds of infected individuals are unique for COVID-19 or not is still a question to be answered. Chang et al. introduced a “CovNet” which uses a transfer learning framework to improve the models’ generalisation. Experimental results show their models’ efficiency by considering a parameter transferring strategy and an embedding incorporation strategy. Akman et al. propose an end-to-end deep neural network model (called “CIdeR”) for exploring the methodological adaptation to new datasets with different modalities. From the experiments, their proposed model can serve across multiple audio types. However, they found that it is difficult to train a common COVID-19 classifier due to the limitations of a joint usage of datasets.

### 2.2 Domestic activity

Audio tagging of domestic activities can provide important information on health and wellbeing. Yang et al. present an explainable tensor network for monitoring domestic activities via audio signals. They indicated that, the combination of the tensor network can reduce the redundancy of the network.

### 2.3 Music and brain

Music therapy appears promising for its non-drug characteristic, specifically for treatment of mental disorders (8). However, the influences of music on the brain are still an open question to be answered. Wei et al. contribute a review on neurocognition for timbre perception. They conclude that, timbre perception is promising in psychological application. Further. Liu et al. studied timbre fusion of Chinese and Western instruments. This bears interest, given that in a recent study, timbre features are found to be strongly associated with the human affective states (9). Next, Miyamoto et al. introduce a meta-learning strategy in a music generation system. More fundamentally, Corona-González et al. presented a study on personalised theta and beta binaural beats for brain entrainment. The conclusion made is that the neural resynchronisation was met with both personalised theta and beta binaural beats whereas there seemed to be no different mental conditions achieved.

### 2.4 Artificial hearing

A disyllabic corpus that could be used to examine the performance of pitch recognition of cochlear implant users was introduced. Wang et al. found that, higher scores of tone recognition tend to be achieved by listeners with longer cochlear implant listening experience.

### 2.5 Speech emotion recognition

Speech emotion recognition is a widely-studied field in affective computing. The combination of task-specific speech enhancement and data augmentation as a strategy has been used for improving the overall multimodal emotion recognition in noisy conditions. This contribution of Kshirsagar et al. can benefit the speech-based affective information retrieval task in real-world applications.

## 3. Insights and perspectives

When reading over the collection of this research topic, one finds promising potential of computer audition that can benefit manifold health-related aspects of our life. However, one needs to fully consider the current limitations and keep an eye on the future progress of computer audition.

First, *data scarcity* is still a serious challenge (10) that constrains the fast development of audio based large models. The hardware limitations and further factors impede the collection of high-quality audio data at large scale which could provide sufficient training for current state-of-the-art large models in this domain. Besides, the annotation of audio data (specifically for medical applications) is often difficult. Therefore, advanced strategies such as meta-learning (11), and self-supervised learning should be taken into account prior to the event of generalist (medical) AI (12).

Second, fundamental studies on features, models, and strategies are of interest but limited. Among this collection, we can see some contributions focus on extracting novel audio features to improve the performance of models. We hope to see more works in the future towards the interpretation of the models (13).

Third, the mechanism of the brain’s perception of audio is worth exploring in considerably more depth. It will not only be beneficial for building brain-inspired deep learning models, but also for our understanding more deeply music/audio therapy.

Last but not the least, how to leverage the power of the coming large models to discover more possibilities of computer audition is an open question to be answered.

## Footnotes

^1^ https://openai.com/blog/new-models-and-developer-products-announced-at-devday
